# Gap junction innexin asymmetry in C. elegans suggests a diode blocking mechanism to prevent antidromic backpropagation from motor neurons to command interneurons

**DOI:** 10.17912/micropub.biology.002326

**Published:** 2026-08-21

**Authors:** John White

**Affiliations:** 1 Emeritus Professor, University of Wisconsin–Madison, Madison, WI, United States

## Abstract

In *C. elegans* , gap junctions between command interneurons and motor neurons mediate rapid behavioural transitions. However, antidromic backpropagation driven by the putative proprioceptive activity of motor neurons with Extended Longitudinal Neurites (ELNs) must be blocked to maintain circuit directionality. Analysing single-cell transcriptomics (CeNGEN), I identify systematic innexin gene expression asymmetry across major motor circuit electrical synapses. Presynaptic interneurons and postsynaptic motor neurons express distinct, non-overlapping innexin subunits. This molecular asymmetry suggests a rectified, diode-like gating mechanism that favours orthodromic signalling while preventing antidromic backpropagation from proprioceptive motor neurons, providing a structural framework for directional electrical transmission.

**Figure 1. Innexin asymmetries in motor neuron circuitry:** Expression asymmetry of gap junction innexins suggests a rectifying diode isolation mechanism between command interneurons and motor circuit processes. **(A) Innexin expression matrix in identified neurons.** Single-cell RNA-seq expression levels (mean TPM, CeNGEN adult dataset; Taylor et al., 2021) of innexins ( *unc-7* , *unc-9* , *inx-21* , *inx-22* , *eat-5* , and *inx-19* ) in head motor neurons (SMB, SMD), body motor neurons (VA, VB), and associated interneurons (AVA, AVB, RIB, SAA). Gray boxes highlight dominant expression or key asymmetrical ratios. Note the pronounced *unc-7* / *unc-9* expression ratio skew toward command interneurons (AVA: 6.15; AVB: 5.65; SAA: 3.87). The mean *unc-7* / *unc-9* ratio for all co-expressing neurons in the organism is 1.60. AVB exhibits a high *unc-7* / *unc-9* ratio together with selective expression of the asymmetric innexin *inx-19* . SAA also exhibits a high *unc-7* / *unc-9* ratio together with selective expression of the asymmetric innexin *eat-5* . **(B) Putative electrical synapses in body motor neuron circuitry.** Schematic representation of asymmetric gap junctions between command interneurons (AVA, AVB) and ventral cord motor neurons (VA, VB). Heterotypic coupling between UNC-7 (interneuron side) and UNC-9 (motor neuron side), as well as selective INX-19 expression in AVB, suggests the presence of functional electrical diodes (indicated by diode symbols) configured to pass depolarizing current anterograde while blocking antidromic back-propagation (Starich et al., 2009; Liu et al., 2020). Green bars indicate the presence of distal, uninnervated process extensions hypothesized to function as proprioceptive stretch receptors (White et al., 1986; Wen et al., 2012). AVA also makes chemical synapses onto VA motor neurons (indicated by arrow). **(C) Putative electrical synapses in head motor neuron circuitry.** Circuit diagram showing asymmetrical gap junctions between head motor neurons (SMB, SMD) and head interneurons (RIB, SAA). EAT-5 is selectively expressed in SMB and SAA, while both INX-21 and INX-22 are selectively expressed in SMD, establishing distinct asymmetric electrical coupling in head motor neuron circuitry (Starich et al., 1996; Simonsen et al., 2014). RIB is a major hub interneuron of the central nervous system (White et al., 1986; Cook et al., 2019). The rectifying electrical connections to SMB and SMD act to isolate RIB from the back-propagation of local electrical activity produced by the proprioceptive activation of SMB and SMD. SAA makes a chemical synapse (arrow) onto SMD in addition to its electrical connection to SMB. **(D) Process morphology of body motor neurons VA3 and VB4.** Anatomical schematics depicting the extended, morphologically undifferentiated distal processes (green) of VA3 and VB4 extending beyond neuromuscular junction zones (red bars). **(E) Process morphology of dorsal head motor neurons SMBDL and SMDDL.** Anatomical schematics illustrating the process trajectories of SMBDL and SMDDL, highlighting distal sensory/proprioceptive extensions (green) relative to synaptic output regions within the nerve ring (red). These processes run down the length of the body in small sub-lateral cords. Cell bodies are shown in black. Panels (D) and (E) adapted from White et al. (1986).

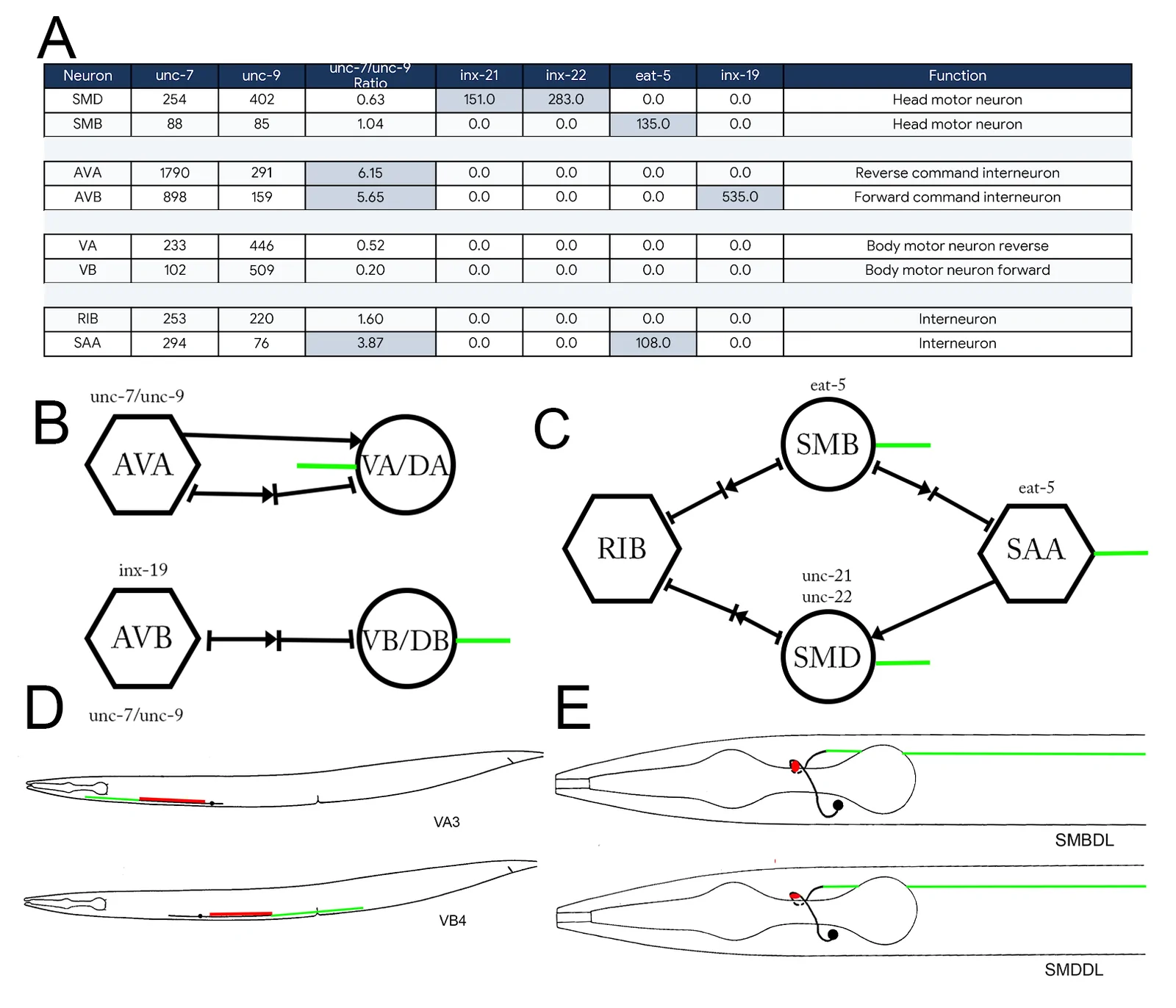



## Description

The nervous system of *C. elegans* comprises a total complement of only 302 neurons, yet contains the foundational circuit principles of vertebrate nervous systems, which, in humans, contain about 86 billion neurons (Goriely, 2024). In the mid-1960s, Sydney Brenner pioneered the use of *C. elegans* as a genetic model system to dissect nervous system development and function (Brenner, 1974). Since then, extensive research has generated comprehensive datasets detailing both the structural connectivity of the nervous system (White et al., 1986; Cook et al., 2019) and single-cell gene expression patterns across development (Taylor et al., 2021). These data are curated in publicly accessible databases such as WormAtlas (Altun et al., 2009) and CeNGEN ( *Caenorhabditis elegans* Neuronal Gene Expression Network) (Taylor et al., 2021).

Gap junctions are specialized ion channels that facilitate electrical coupling between cells (Sohl et al., 2005). They are made up of innexin subunits (connexins in vertebrates), of which there are 25 in *C. elegans* (Starich et al., 1996; Simonsen et al., 2014). In this study, I analyse the expression of innexins in identified motor neurons and command interneurons. Specifically, I examine the spectrum of expressed innexins at coupled electrical synapses, where striking molecular asymmetries suggest these junctions function as rectifying diodes (Starich et al., 2009; Liu et al., 2020).

The expression maps of the 25 *C. elegans* innexins reveal two general patterns: generalized expression, such as *unc-7* and *unc-9* which are co-expressed across 69.9% of all neurons, and restricted expression, where certain innexins exhibit relatively high expression in small neuronal subsets, such as *inx-21* and *inx-22* which are uniquely expressed in SMD neurons (Taylor et al., 2021).

A generic gap junction is broadly considered a symmetrical structure that permits bidirectional passage of ions or small molecules between coupled cells (Sohl et al., 2005). However, functionally asymmetric electrical synapses have been described that preferentially permit current flow in one direction (Phelan et al., 2008; Starich et al., 2009; Liu et al., 2020). Such gap junctions are structurally asymmetric, most straightforwardly achieved by incorporating differing innexin subunits into the paired hemichannels of adjacent cells (Palacios-Prado et al., 2014).

Figure 1A details examples of molecular asymmetry in neural circuits associated with major motor neurons innervating body wall muscles (VA, VB) and head muscles (SMB, SMD). These classes of neuron all have Extended Longitudinal Neurites (ELNs). One prominent form of asymmetry involves the stoichiometric ratio of the widely expressed innexins *unc-7* and *unc-9* . While the mean organism-wide *unc-7* / *unc-9* ratio across co-expressing neurons is 1.60, major command interneurons AVA and AVB exhibit ratios of 6.15 and 5.65, respectively—nearly four times the neuronal baseline. Even more prominent innexin asymmetries are demonstrated by the restricted expression of *inx-21* and *inx-22* in SMD and *eat-5* in SMB and SAA.

Strikingly, these asymmetrical gap junctions selectively involve circuitry tied to motor neurons driving body (VA, VB) and head (SMB, SMD) locomotion ( Fig. 1B, C). All these motor neuron classes share the hallmark anatomical feature of ELNs: morphologically undifferentiated longitudinal processes situated distal to their neuromuscular output zones ( Fig. 1D, E). It has long been posited that these distal extensions act as proprioceptors, transducing local body bending into downstream muscle activation during undulatory locomotion (White et al., 1986; Wen et al., 2012).

Rectifying gap junctions function effectively as blocking diodes that restrict ionic current flow to a single direction (Shui et al., 2020). For motor neurons possessing dual motor-proprioceptive modalities (VA, VB, SMB, SMD), diode rectification would be vital to prevent locally generated, stretch-activated electrical signals from back-propagating antidromically into critical central interneurons, such as the major network hub RIB ( Fig. 1C ). Supporting this hypothesis, major motor neurons lacking extended proprioceptive process extensions, such as RMD, exhibit no corresponding innexin asymmetries in their gap junctions.

Combining connectomic mapping with cell-specific transcriptomic profiling can provide key functional insights into neural circuitry. Such findings provide testable hypotheses to guide future electrophysiological experiments and computational modelling aimed at deciphering how a nervous system generates the behaviours that control an organism.

## Methods

**Transcriptomic and Connectomic Data Analysis**

Single-cell RNA sequencing expression levels (Transcripts Per Million, TPM) for all 25 *Caenorhabditis elegans* innexins across 133 identified neuronal cell types were extracted from the adult CeNGEN dataset (Taylor et al., 2021). Co-expression frequencies and mean expression ratios were calculated using custom Python scripts running in Google Colab (Python 3.10; pandas v2.0, numpy v1.24). Neuronal co-expression of *unc-7* and *unc-9* was defined as non-zero TPM values (>0.0 TPM) in both genes across the 133 annotated neuron classes. Anatomical connectivity, chemical and electrical synapse counts, and motor neuron process morphologies were cross-referenced against the EM connectome datasets (White et al., 1986; Cook et al., 2019) and WormAtlas (Altun et al., 2009).
